# Supplementary material for: Direct Photochemical Synthesis of Substituted Benzo[b]fluorenes
Source: Org Lett. 2024 Nov 25;26(48):10364–8. doi: 10.1021/acs.orglett.4c03978 (PMC11629382; doi:10.1021/acs.orglett.4c03978)
Supplement: Supplementary file 2 — ol4c03978_si_002.zip [file ol4c03978_si_002.zip › FID for Publication/4q/20240720_RC-2-C-4-130A_01/plots/RC-2-C-4-130A_FLUORINE_20240720_01_plot01.pdf]

RC-2-C-4-130A

Location	**55**	Pulse sequence	**FLUORINE**	Temperature	**25**	Suboperator	**RuairiCrawford**
Date collected	**2024-07-20**	Solvent	**cdcl3**	Spectrometer	**newucd400-vnmrs400**	Operator	**MBaumann**

SAMPLE

date	**Jul 20 2024**
solvent	**cdcl3**
file	**/home/data/MBaumann/ucd400/RuairiCrawford/2024/20240720_RC-2-C-4-130A_01/RC-2-C-4-130A_FLUORINE_20240720_01.fid**

ACQUISITION

sw	**89285.7**
at	**1.000**
np	**178572**
fb	**47800**
bs	**2**
ss	**2**
d1	**1.000**
nt	**32**
ct	**32**

TRANSMITTER

tn	**F19**
sfrq	**376.230**
tof	**9882.0**
tpwr	**60**
pw	**5.000**

DECOUPLER

dn	**C13**
dof	**0**
dm	**nnn**
decwave	**W40_P8012**
dpwr	**34**
dmf	**29412**

PRESATURATION

satmode	**n**
wet	**n**

SPECIAL

temp	**25.0**
gain	**60**
spin	**0**
hst	**0.008**
pw90	**15.000**
alfa	**10.000**

FLAGS

il	**n**
in	**n**
dp	**y**
hs	**nn**

PROCESSING

lb	**0.50**
fn	**262144**

DISPLAY

sp	**-76625.1**
wp	**89285.7**
rfl	**76625.1**
rfp	**0**
rp	**4.0**
lp	**0**

PLOT

wc	**251**
sc	**8**
vs	**225**
th	**1**

ai cdc ph

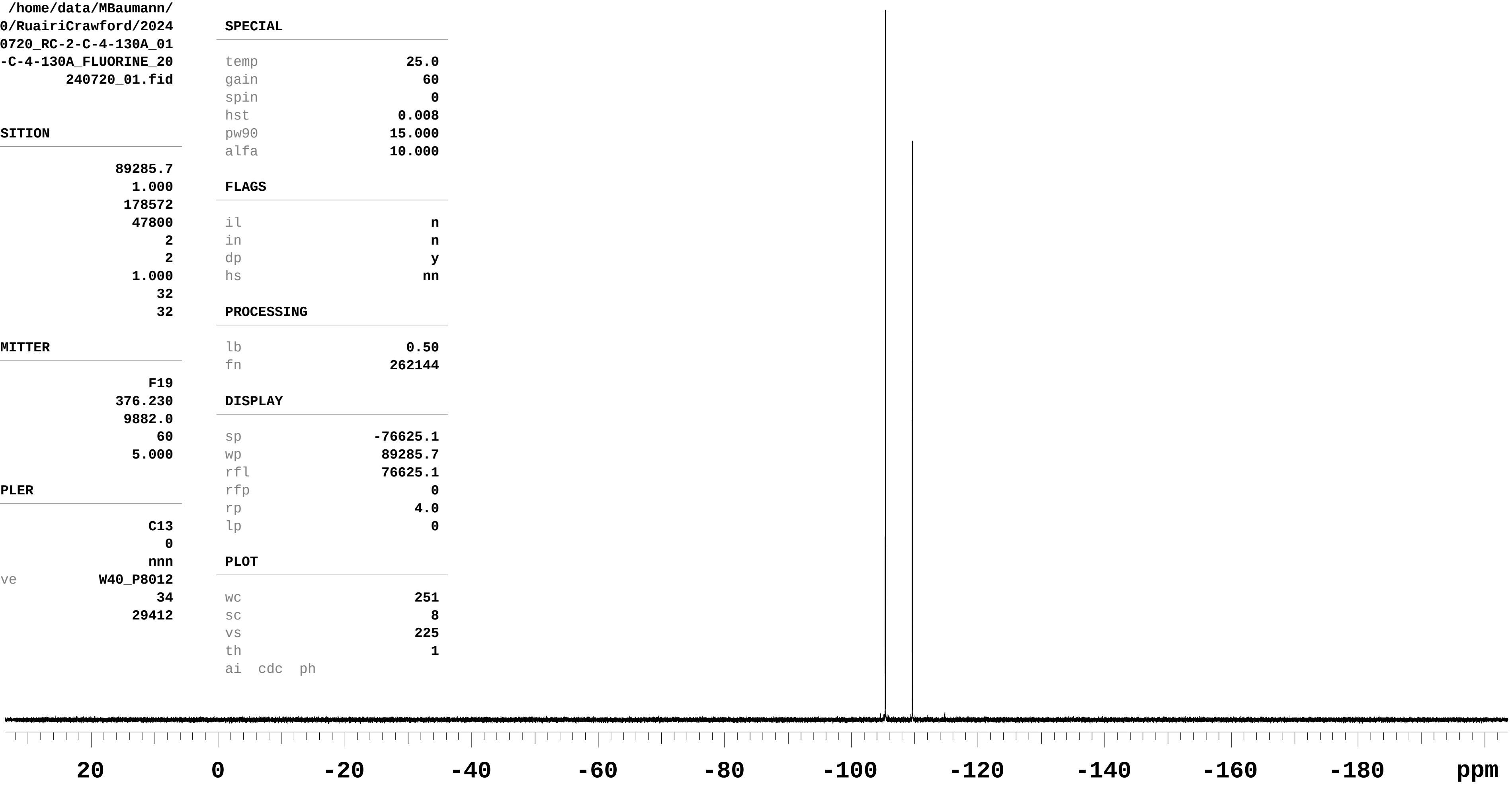

Plotname: RC-2-C-4-130A_FLUORINE_20240720_01_plot01

Data file /home/data/MBaumann/ucd400/RuairiCrawford/2024/20240720_RC-2-C-4-130A_01/RC-2-C-4-130A_FLUORINE_20240720_01.fid

Plot date 2024-07-20
